# Military and demographic predictors of mental ill-health and socioeconomic hardship among UK veterans

**DOI:** 10.1186/s12888-021-03296-x

**Published:** 2021-07-06

**Authors:** H. Burdett, N. T. Fear, S. Wessely, R. J. Rona

**Affiliations:** King’s Centre for Military Health Research, Department of Psychological Medicine, King’s College London, London, UK

**Keywords:** Veterans, Veterans health, PTSD, Unemployment, Alcohol abuse

## Abstract

**Background:**

Around 8% of the UK Armed Forces leave in any given year, and must navigate unfamiliar civilian systems to acquire employment, healthcare, and other necessities. This paper determines longer-term prevalences of mental ill health and socioeconomic outcomes in UK Service leavers, and how they are related to demographic factors, military history, and pre-enlistment adversity.

**Methods:**

This study utilised data from a longitudinal sample of a cohort study UK Armed Forces personnel since 2003. A range of self-reported military and sociodemographic factors were analysed as predictors of probable Post-Traumatic Stress Disorder, common mental disorders, alcohol misuse, unemployment and financial hardship. Prevalences and odds ratios of associations between predictors and outcomes were estimated for regular veterans in this cohort.

**Results:**

Veteran hardship was mostly associated with factors linked to socio-economic status: age, education, and childhood adversity. Few military-specific factors predicted mental health or socio-economic hardship, except method of leaving (where those leaving due to medical or unplanned discharge were more likely to encounter most forms of hardship as veterans), and rank which is itself related to socioeconomic status.

**Conclusion:**

Transition and resettlement provisions become increasingly generous with longer service, yet this paper shows the need for those services becomes progressively less necessary as personnel acquire seniority and skills, and instead could be best targeted at unplanned leavers, taking socioeconomic status into consideration. Many will agree that longer service should be more rewarded, but the opposite is true if provision instead reflects need rather than length of service. This is a social, political and ethical dilemma.

## Background

Around 15,000 (8%) members of the UK Armed Forces leave the Services each year [1]. Most veterans (defined in the UK as those who have served for at least 1 day) need to find new occupations, as well as access health and other civilian services; such transitions might be challenging [2]. Costs to the UK of poor transition are estimated to be over £100million [3].

The UK Ministry of Defence (MoD), in partnership with a civilian service provider, aims to facilitate such transitions through the resettlement process [4]. The MoD evaluate the effectiveness of the resettlement process by following up individuals 6 months after leaving: employment rates for veterans who utilise the service are comparable to civilians, but despite the resettlement process a substantial proportion are unemployed within this six-month follow-up window: 7% of leavers 2017/2018 are unemployed, rising to 13% among those medically discharged [5]). However, there is a paucity of research regarding longer-term employment outcomes among UK Service leavers.

Mental health disorders make up 6% of claims to the Armed Forces Compensation Scheme (which compensates ex-service personnel and dependents where illness, injury or death was caused by service since April 2005), but 15% of all Guaranteed Income Payments (the highest level of award) [6]. This indicates that, while new high-level awards for mental health conditions have dropped in recent years, post-service mental health continues to be a serious challenge. There is a paucity of research on which pre-service, service-related and other demographic factors are associated with increasing risk of poor mental health and socioeconomic outcomes. Employment and financial stability are associated with mental health in the general population [7], and employment may be a key part of the recovery pathway for veterans with mental health difficulties [8].

The universal healthcare provided by the UK National Health Service and otherwise fairly comprehensive state benefits have produced a system whereby, after a period of MoD-supported transition, veterans’ needs have been met primarily through the civilian sector (albeit with veteran-specific initiatives embedded within civilian welfare systems). It is only recently that the state has taken a larger, more direct role in veterans’ welfare through such new initiatives as the Office for Veterans’ Affairs [9]. Other nations differ in their definition of “veteran” qualifying for post-service support [10], and in the nature of post-service support; for example the US provides healthcare, funding for higher education, and other forms of support to eligible veterans through such institutions as the US Veterans Administration. Thus the US has opted to treat veterans as a separate population from other members of the population whereas UK policy has, at least until recently, seen veterans as civilians. Consequently, it is difficult to make comparisons between countries, due to different provisions for transition, resettlement, and remuneration for those leaving the Armed Forces in general and for those with physical or mental disorders, and hence findings from other countries may not be generalisable.

King’s Centre for Military Health Research (KCMHR) began following a cohort of UK Armed Forces Personnel from the outbreak of the 2003 Iraq War and has collected mental health measures and socioeconomic data for these personnel (and others recruited subsequently) over a period of 13 years [11–13]. This dataset now includes details of nearly 5000 veteran members of the cohort who have left the UK Armed Forces since 2004. Using these data, we:

1. Assessed the longer-term prevalence rates of mental ill health and adverse socioeconomic outcomes in terms of unemployment and financial difficulties
2. Determined how demographic factors, pre-enlistment adversity, military history, and sociodemographic factors give rise to increased risk of unemployment and financial difficulties among UK Service leavers.

## Methods

### Participants

Data collection for the first phase of the KCMHR cohort study began with the commencement of the Iraq War in 2003 [11]. Participants were identified by the UK Ministry of Defence’s Defence Statistics unit. A list of all personnel, excluding special forces and high security personnel, who had deployed on to Iraq between Jan 18 and April 28, 2003, was generated. A similar list of UK service personnel serving in the armed forces on March 31, 2003, but not deployed to Iraq was generated as the comparison group. The total sampling frame comprised 17,499 individuals. Several strategies were used to contact potential participants. Initially study participants were allocated either to receive a questionnaire by post, or, where military postcodes indicated a large number of personnel at the same location, serving personnel were assigned a visit from the research team. Visits and mail-outs were done simultaneously. Those who did not wish to participate during a base visit could leave at any time. Data collection was performed June 2004–March 2006, with 10,272 responding, a response rate of 59% (62% among the deployed group and 56% in the non-deployed group). Full details of sample identification and data gathering procedures can be found elsewhere [11].

Data collection for the second phase was performed from Nov 2007 to Sep 2009, and included those respondents at the first phase who were still eligible for contact (*n* = 9395), as well as a new sample of those who had deployed to the conflict in Afghanistan (*n* = 1789) as well as a new sample of non-deployed controls (*n* = 6628). 9990 (56%) responded at phase 2; response rates were 68% for the follow-up sample, 50% for the new respondents who had deployed to Afghanistan and 40% for the new non-deployed controls [12].

Phase 3 data was collected between Oct 2014-Dec 2016. Those who consented to further contact were sent questionnaires (*n* = 12,280), along with an additional sample of non-deployed comparators (*n* = 8581). 8093 (44%) responded on this third phase; of these 58% responded in the follow-up sample from which most of the participants in this study are drawn (response among the replenishment sample was 24%) [13].

The samples were representative of the sampling frames available from the Defence Statistics, excluding those in Special Forces. The veterans within this study may have left at any time over the course of these data collection phases. To maximise the available data, we used the most recently-provided data for each individual; thus relationships between outcome and exposure factors are effectively cross-sectional, except where data is missing and is being imputed from last available data. This study focuses on ex-regular veterans, and hence those still serving at last response were excluded; furthermore both those who were serving as a reserve, or were no longer serving but had only served as a reserve, were excluded as their civilian career history differs from regulars. In total this study includes 3095 participants who responded in the most recent phase as well as 896 who last responded at phase 2 and 301 who last responded at phase 1, for a total of 4292 ex-Service personnel in the study.

### Outcomes

Mental health measures were acquired via self-report questionnaire for all phases. Probable PTSD was determined using the 17-item National Centre for PTSD Checklist (PCL-C). PCL-C comprises 17 items each rated on a 5-point scale score 1–5 (range 17–85). We used the recommended cutoff of 50 or more to determine caseness. At this cutoff the PCL has an overall diagnostic efficiency of 0.825 as verified against the gold standard Clinician-Administered PTSD Scale. Cronbach’s alpha for the scale is 0.939 [14].

Common mental disorders (CMD) were measured using the 12-item General Health Questionnaire (GHQ-12). Each of the 12 items provides four options for response of increasing severity; these were scored 0–0–1-1, using the commonly-used cutoff of 4 to qualify as a case (range 0–12). Cronbach’s alpha for GHQ-12 has been estimated as being between 0.82 and 0.86 [15].

Alcohol misuse was determined using the World Health Organization Alcohol Use Disorders Identification Test [16], which includes three items on alcohol intake, three on alcohol dependence and four on alcohol-related problems, for a total score range of 0–40. This study used a relatively high cutoff of 16 (defined as hazardous use that is likely to be harmful to health), as is customary in studies of this cohort due to the high level of drinking in the UK military [17].

Socio-economic outcomes were also taken from self-report questions. Questionnaires asked whether respondents were currently working in a civilian job, as a private security contractor, self-employed, not working and looking for employment, not working due to ill health, or retired. Those not working (whether due to ill health or looking for employment) were classified as unemployed (including a small number who were over 65 but did not state they were retired); those who indicated some form of employment (including self-employed) were classified as employed; those retired, in education, or in some other non-paying role were included in a third category. For brevity this last category is not discussed in this article, but data is available on request.

Questionnaires asked respondents how they are managing financially; a five-point scale was provided comprising “living comfortably”, “doing alright”, “just about getting by”, “finding it quite difficult” and “finding it very difficult”. This was reduced to a binary variable which defined financial difficulty as those endorsing “just about getting by”, “finding it quite difficult” or “finding it very difficult”. This question was only asked in the last phase of data collection, hence findings for this variable apply only to those most recent respondents (*N* = 3007).

### Covariates

Age was determined at time of most recent questionnaire response and subdivided into 5- or 10-year brackets. We also include variables for last reported education (divided into four categories: no qualifications, O-level or equivalent, A-level or equivalent, and degree level or higher), and childhood adversity in two domains: childhood externalising behaviour and family adversity.

Two separate variables for childhood externalising behaviour and family adversity were derived from a 16-item checklist using the root “When I was growing up …” [18]. This was an original checklist based on a combination of three items adapted from the Adverse Childhood Exposure scale [19] and other items based on general population evidence of childhood exposures for later adverse health outcomes; the checklist has not been validated as a measure, nor have either of the derived variables. Family adversity score was determine by summing endorsements to not coming from a close family, getting shouted at a lot at home, not feeling valued by family, regularly witnessing fighting or verbal abuse between parents, not having a family member they could talk to, being regularly hurt by a parent/caregiver, having a parent with substance abuse problems, and/or not doing things as a family [20]. Those who endorsed getting into fights at school plus at least one of playing truant from school, being suspended or expelled from school, or doing things that got them into trouble with the police were classified as demonstrating childhood externalising behaviour [21].

Military-related factors included rank, Service arm, role, history of deployment, and method of leaving the Services. Rank was determined from the most recent response, and categorised into Commissioned Officer, Senior Non-Commissioned Officer (SNCO) (sergeant-equivalent rank and above), Junior Non-Commissioned Officer (JNCO) (lance-corporal and corporal-equivalent ranks), and other ranks (i.e. private soldiers and equivalent enlisted ranks). Service arm was separated into Army, Royal Air Force (RAF), and Naval Services (which includes Royal Marines). Deployment was separated into those who had deployed to Iraq and/or Afghanistan at least once, and those who had deployed to neither. Role in parent unit (a small proportion of individuals may have different roles on different deployments) was reduced to those with a combat role, and all other roles. Method of leaving was determined by self-report; “End of service term or run out date”, “premature voluntary release”, and “voluntary redundancy” were categorised as “planned leaving”, “administrative discharge”, “temperamental unsuitability”, “disciplinary discharge” and “compulsory redundancy” were categorised as “unplanned leaving”, and “medical discharge” was treated as a third category.

### Analysis

Individuals may have provided multiple responses to the same questions in different phases of data collection. Consequently, responses were taken at the latest phase in which they became a veteran (i.e. most recent post-transition responses). Where data for socio-demographic and military factors were missing, prior responses were utilised, but this was not done for outcome variables to ensure that only contemporaneous transition outcomes were analysed.

There were excellent completion rates for most variables (< 0.5% missing data for most variables, < 5% missing data for education achievement, PCL, GHQ, AUDIT, and socioeconomic outcomes), therefore analyses involving these variables was performed on a complete-case basis.

Associations between mental health/socioeconomic outcomes and demographic/military factors were determined by logistic regression, producing odds ratios (OR) as indicators of effect size. For employment status, multinomial logistic regression (logistic regression with more than two discrete outcomes) was utilised to produce multinomial odds ratios (mOR). Subsequent to the univariate analysis, an adjusted model was produced in which all factors associated with each outcome were included as potential confounders to take account of non-independence between covariates.

The underlying cohort was randomly sampled from the serving population of the UK Armed Forces and weighted for both sample and response; no weights were applied as the veterans in this article represent a non-random subsample. Analysis was performed using STATA 15 [22].

## Results

### Demographics and military variables

Table 1 shows the distributions of the demographic and military variables. Most of the veterans included in this sample are over 40 years old, male, served in the Army, were senior non-commissioned officers, left in a planned way, and attained A-level or higher qualifications.

**Table 1:** Frequencies and proportions of demographic and military factors

Factor	Category	Number (*N* = 4292)	%
**Sociodemographic factors**			
Sex	Male	3837	89.4
	Female	455	10.6
Age at response (years)	< 25	219	5.1
	25–29	380	8.9
	30–34	641	14.9
	35–39	397	9.3
	40–49	1606	37.4
	50+	1049	24.4
Education	No qualifications	240	5.8
	O-levels or equivalent	1159	28.0
	A-levels or equivalent	1421	34.3
	Degree or higher	1319	31.9
Childhood family adversity	0/1	2669	65.0
	2/3	780	19.0
	4+	660	16.1
Childhood externalising behaviour	No	3447	83.3
	Yes	693	16.7
**Military factors**			
Rank^a^	Officer	883	20.7
	SNCO	1576	36.9
	JNCO	993	23.2
	Other rank	825	19.3
Service arm	Naval Services	762	17.8
	Army	2642	61.6
	RAF	888	20.7
Deployment to Iraq/Afghanistan	Did not deploy	1539	35.9
	Deployed	2753	64.1
Role in parent unit	Combat support/CSS	3066	71.8
	Combat	1203	28.2
Method of leaving	Planned	3508	89.1
	Unplanned	157	4.0
	Medical	272	6.9

^a^*“SNCO”* Senior Non-Commissioned Officer, *“JNCO”* Junior Non-Commissioned Officer

### Mental health and socioeconomic outcomes

Mental health and socioeconomic outcomes are shown in Table 2. CMD is the most frequent negative outcome encountered by veterans, but a sizeable percentage experience PTSD, alcohol misuse, financial difficulties and unemployment, ranging from 7.4 to 11.8%.

**Table 2:** Posttraumatic stress disorder (PTSD), common mental disease (CMC) alcohol misuse and socioeconomic outcomes at last assessment

Outcome	Value	Number (*N* = 4292)	%
Probable PTSD	Case	329	7.8
CMD	Case	990	23.5
Alcohol misuse	Case	488	11.8
Employment status	Employed	3288	82.3
	Unemployed	297	7.4
	Retired/education/other	412	10.3
Financial difficulty	Living comfortably/ doing all right	2406	80.0
	Just getting by/finding it difficult	601	20.0

### Associations between demographic and service-related factors with mental health and socioeconomic outcomes

Significance and effect size of the associations between demographics, military-related factors, and post-service mental health of veterans are shown in Table 3. After adjustment, probable PTSD was associated with childhood adversity, lacking any formal qualifications, being ex-Army, lower rank, deployment to Afghanistan/Iraq and having a combat role. The largest impact was from leaving the Armed Forces due to medical discharge; other unplanned leaving, rank and childhood adversity also had substantial effect sizes.

**Table 3:** Associations between demographic and service-related factors by mental health outcomes

Probable PTSD (*N* = 4211)				
Factor	Value			
		Number (%)	OR (95% CI)	AOR (95% CI)^a^
Sex	Male	298 (7.9)	1	–
	Female	31 (7.0)	0.87 (0.59–1.28)	–
Age	< 25	33 (15.4)	2.57 (1.69–3.91)***	0.65 (0.35–1.21)
	25–29	44 (11.6)	1.86 (1.28–2.69)**	0.73 (0.43–1.24)
	30–34	74 (11.7)	1.87 (1.37–2.56)***	0.76 (0.49–1.19)
	35–39	42 (10.9)	1.71 (1.18–2.50)**	1.00 (0.62–1.60)
	40–49	104 (6.6)	1	1
	50+	32 (3.1)	0.45 (0.30–0.68)***	0.68 (0.42–1.09)
Education	No qualifications	38 (16.5)	2.49 (1.67–3.72)***	1.84 (1.14–2.99)*
	O-levels/ equivalent	107 (9.5)	1.32 (0.99–1.74)	1.02 (0.73–1.43)
	A-levels/ equivalent	103 (7.4)	1	1
	Degree or higher	62 (4.8)	0.63 (0.45–0.87)**	0.92 (0.62–1.38)
Childhood family adversity	0/1	141 (5.4)	1	1
	2/3	57 (7.5)	1.42 (1.03–1.95)*	1.29 (0.90–1.86)
	4+	109 (16.7)	3.54 (2.71–4.62)***	2.90 (2.11–4.00)***
Childhood externalising behaviour	No	198 (5.8)	1	1
	Yes	116 (17.1)	3.32 (2.59–4.24)***	1.46 (1.07–2.00)*
Rank	Officer	32 (3.7)	0.73 (0.48–1.11)	1.01 (0.60–1.69)
	Senior NCO	77 (5.0)	1	1
	Junior NCO	102 (10.5)	2.24 (1.65–3.05)***	1.93 (1.28–2.91)**
	Other rank	114 (14.1)	3.12 (2.31–4.23)***	3.17 (1.94–5.18)***
Service arm	Naval Services	49 (6.5)	0.66 (0.48–0.91)*	0.58 (0.39–0.87)**
	Army	246 (9.5)	1	1
	RAF	34 (3.9)	0.38 (0.27–0.55)***	0.55 (0.36–0.84)**
Deployment to Iraq/Afghanistan	Did not deploy	84 (5.5)	1	1
	Deployed	245 (9.1)	1.71 (1.32–2.21)***	1.52 (1.11–2.07)**
Role in parent unit	Combat support/CSS	177 (5.9)	1	1
	Combat	148 (12.5)	2.29 (1.82–2.88)***	1.52 (1.14–2.03)**
Method of leaving	Planned	186 (5.4)	1	1
	Unplanned	28 (18.0)	3.83 (2.48–5.92)***	2.16 (1.31–3.58)**
	Medical	79 (29.5)	7.32 (5.42–9.90)***	6.79 (4.80–9.62)***
Common Mental Disorders (*N* = 4217)				
Factor	Value	Number (%)	OR (95% CI)	AOR (95% CI)^a^
Sex	Male	891 (23.6)	1	–
	Female	99 (22.3)	0.93 (0.73–1.17)	–
Age	< 25	76 (36.2)	1.87 (1.38–2.53)***	1.05 (0.68–1.63)
	25–29	120 (31.7)	1.53 (1.19–1.95)**	1.02 (0.72–1.44)
	30–34	169 (26.7)	1.20 (0.97–1.49)	0.81 (0.61–1.08)
	35–39	102 (26.2)	1.17 (0.91–1.51)	0.88 (0.65–1.20)
	40–49	367 (23.3)	1	1
	50+	156 (15.1)	0.59 (0.48–0.72)***	0.71 (0.56–0.90)**
Education	No qualifications	69 (29.9)	1.43 (1.05–1.95)*	1.17 (0.82–1.67)
	O-levels/ equivalent	279 (24.8)	1.11 (0.92–1.33)	0.95 (0.77–1.17)
	A-levels/ equivalent	321 (22.9)	1	1
	Degree or higher	274 (20.9)	0.89 (0.74–1.07)	1.17 (0.94–1.46)
Childhood family adversity	0/1	508 (19.3)	1	1
	2/3	205 (26.8)	1.52 (1.26–1.84)***	1.50 (1.22–1.84)***
	4+	226 (34.7)	2.22 (1.84–2.68)***	1.89 (1.52–2.35)***
Childhood externalising behaviour	No	703 (20.7)	1	1
	Yes	250 (36.8)	2.23 (1.87–2.66)***	1.42 (1.14–1.76)**
Rank	Officer	157 (18.0)	0.94 (0.76–1.17)	1.09 (0.84–1.41)
	Senior NCO	292 (18.9)	1	1
	Junior NCO	264 (27.1)	1.60 (1.32–1.93)***	1.46 (1.14–1.88)**
	Other rank	270 (33.3)	2.15 (1.77–2.60)***	1.80 (1.32–2.46)***
Service arm	Naval Services	163 (21.7)	0.82 (0.67–0.99)*	0.89 (0.71–1.11)
	Army	654 (25.3)	1	1
	RAF	173 (19.7)	0.72 (0.60–0.87)**	0.94 (0.75–1.18)
Deployment to Iraq/Afghanistan	Did not deploy	328 (21.6)	1	1
	Deployed	662 (24.5)	1.18 (1.01–1.37)*	1.13 (0.94–1.34)
Role in parent unit	Combat support/CSS	642 (21.3)	1	1
	Combat	341 (28.8)	1.50 (1.29–1.75)***	1.13 (0.93–1.36)
Method of leaving	Planned	687 (19.9)	1	1
	Unplanned	58 (37.2)	2.38 (1.70–3.33)***	1.80 (1.25–2.60)**
	Medical	134 (50.0)	4.02 (3.12–5.18)***	3.74 (2.84–4.91)***
Alcohol misuse (N = 4,125)				
Factor	Value			
		Number (%)	OR (95% CI)	AOR (95% CI)^b^
Sex	Male	465 (12.6)	1	1
	Female	23 (5.2)	0.38 (0.25–0.58)***	0.45 (0.27–0.75)**
Age	< 25	61 (28.6)	3.67 (2.61–5.16)***	2.54 (1.53–4.22)***
	25–29	81 (22.9)	2.71 (2.01–3.66)***	2.17 (1.42–3.30)***
	30–34	89 (14.5)	1.55 (1.17–2.05)**	1.43 (1.00–2.07)
	35–39	38 (10.0)	1.01 (0.70–1.47)	0.91 (0.58–1.42)
	40–49	152 (9.9)	1	1
	50+	67 (6.6)	0.64 (0.48–0.86)**	0.70 (0.50–0.99)*
Education	No qualifications	44 (19.1)	1.98 (1.37–2.86)***	1.44 (0.94–2.21)
	O-levels/ equivalent	181 (16.1)	1.60 (1.27–2.02)***	1.31 (1.01–1.72)*
	A-levels/ equivalent	149 (10.7)	1	1
	Degree or higher	105 (8.1)	0.73 (0.56–0.95)*	1.08 (0.79–1.48)
Childhood family adversity	0/1	230 (8.9)	1	1
	2/3	121 (16.2)	1.97 (1.56–2.50)***	1.74 (1.34–2.26)***
	4+	114 (18.2)	2.27 (1.78–2.90)***	1.64 (1.24–2.17)**
Childhood externalising behaviour	No	300 (9.0)	1	1
	Yes	174 (26.4)	3.61 (2.93–4.46)***	2.28 (1.77–2.92)***
Rank	Officer	66 (7.6)	0.87 (0.64–1.18)	1.05 (0.73–1.52)
	Senior NCO	131 (8.7)	1	1
	Junior NCO	135 (14.3)	1.75 (1.36–2.26)***	1.12 (0.79–1.57)
	Other rank	152 (19.1)	2.48 (1.93–3.19)***	1.09 (0.72–1.65)
Service arm	Naval Services	74 (10.1)	0.70 (0.53–0.91)**	0.99 (0.73–1.50)
	Army	350 (13.8)	1	1
	RAF	64 (7.4)	0.50 (0.38–0.66)***	0.79 (0.58–1.10)
Deployment to Iraq/Afghanistan	Did not deploy	160 (10.7)	1	–
	Deployed	328 (12.5)	1.19 (0.97–1.45)	–
Role in parent unit	Combat support/CSS	293 (9.9)	1	1
	Combat	189 (16.4)	1.78 (1.47–2.17)***	1.18 (0.93–1.50)
Method of leaving	Planned	367 (10.7)	1	1
	Unplanned	46 (29.7)	3.53 (2.48–5.06)***	2.19 (1.44–3.31)***
	Medical	43 (16.3)	1.63 (1.15–2.29)**	1.29 (0.88–1.88)

* *p* < 0.05 ** *p* < 0.01 *** *p* < 0.001

^a^Adjusted for all factors except sex

^b^Adjusted for all factors

Some of these associations held for CMD, which was associated with lower rank, childhood adversity, and unplanned leaving, particularly those with medical discharges. Deployment, role, and Service arm were not associated with veteran CMD. The oldest veterans were less likely to report symptoms of CMD.

There was an inverse relationship between age and alcohol misuse, and men were more likely to misuse alcohol than women. Those educated to O-level standard were more likely to misuse alcohol than those who were more educated, and those who experienced childhood adversity were more likely to misuse alcohol. Military factors were not associated with alcohol misuse except that alcohol misuse was associated with unplanned leaving, but not with medical discharges.

Associations between demographic/service-related factors and socioeconomic outcomes are shown in Tables 3 and 4. Young veterans were more likely to be unemployed, but there was no overall trend with respect to age and financial difficulty. Those with higher educational attainment were less likely to be unemployed or experience financial difficulty. Those with a history of childhood adversity were more likely to encounter financial hardship after leaving service, but not unemployment. Service arm did not affect unemployment or financial difficulty, but higher ranks were less likely to encounter financial hardship (though rank did not make a difference to likelihood of unemployment). As with mental health outcomes, the largest impact was due to method of leaving, where again medical dischargees were the most at-risk group of unemployment and financial difficulty.

**Table 4:** Associations between demographic and service-related factors and employment outcomes (*N* = 4292)

Factor	Value	Unemployed (%)	mOR unemployment (95% CI)	mOR unemployment^a^ (95% CI)	Financial difficulty (%)	OR (95% CI)	AOR (95% CI)^b^
Sex	Male	263 (7.4)	1	1	546 (20.2)	1	
	Female	34 (8.1)	1.22 (0.84–1.78)	1.20 (0.74–1.94)	55 (17.8)	0.85 (0.63–1.16)	
Age	< 25	40 (19.6)	5.61 (3.69–8.55)***	2.74 (1.46–5.15)**	7 (28.0)	1.42 (0.59–3.44)	0.48 (0.16–1.41)
	25–29	37 (10.8)	2.07 (1.38–3.09)***	1.16 (0.66–2.03)	30 (30.3)	1.59 (1.01–2.49)*	0.72 (0.42–1.26)
	30–34	44 (7.4)	1.23 (0.85–1.78)	0.75 (0.46–1.22)	120 (26.7)	1.33 (1.04–1.71)*	0.70 (0.51–0.97)*
	35–39	26 (7.2)	1.17 (0.75–1.84)	0.79 (0.47–1.35)	62 (22.0)	1.03 (0.75–1.41)	0.81 (0.56–1.16)
	40–49	95 (6.3)	1	1	264 (21.5)	1	1
	50+	55 (5.6)	1.06 (0.75–1.50)	1.30 (0.88–1.93)	118 (12.8)	0.54 (0.42–0.68)***	0.75 (0.57–0.98)*
Education	No qualifications	35 (15.7)	2.58 (1.69–3.93)***	2.29 (1.41–3.72)**	44 (31.0)	1.66 (1.13–2.44)*	1.51 (0.97–2.33)
	O-levels/ equivalent	111 (10.0)	1.49 (1.12–1.98)**	1.20 (0.86–1.67)	189 (26.9)	1.36 (1.09–1.70)**	1.31 (1.02–1.67)*
	A-levels/ equivalent	96 (7.1)	1	1	221 (21.3)	1	1
	Degree or higher	50 (4.0)	0.55 (0.38–0.78)**	0.63 (0.42–0.94)*	146 (13.1)	0.56 (0.44–0.70)***	0.89 (0.69–1.16)
Childhood family adversity	0/1	156 (5.8)	1	1	340 (17.8)	1	1
	2/3	62 (8.0)	1.40 (1.03–1.91)*	1.33 (0.95–1.86)	111 (20.3)	1.17 (0.92–1.49)	1.04 (0.81–1.35)
	4+	56 (8.5)	1.53 (1.11–2.11)*	1.15 (0.79–1.65)	119 (27.7)	1.77 (1.39–2.26)***	1.45 (1.11–1.89)**
Childhood externalising behaviour	No	197 (5.7)	1	1	435 (17.5)	1	1
	Yes	84 (12.1)	2.21 (1.68–2.90)***	1.32 (0.94–1.86)	142 (33.4)	2.36 (1.88–2.96)***	1.55 (1.20–2.01)**
Rank	Officer	38 (4.5)	0.90 (0.61–1.34)	1.34 (0.86–2.11)	62 (8.4)	0.44 (0.32–0.59)***	0.56 (0.40–0.78)**
	Senior NCO	83 (5.6)	1	1	214 (17.3)	1	1
	Junior NCO	80 (8.9)	1.64 (1.19–2.25)**	1.25 (0.83–1.90)	186 (29.8)	2.03 (1.62–2.54)***	1.87 (1.42–2.46)***
	Other rank	92 (12.1)	2.54 (1.86–3.48)***	1.42 (0.85–2.38)	139 (33.8)	2.44 (1.90–3.14)***	2.39 (1.67–3.42)***
Service arm	Naval Services	58 (8.1)	1.06 (0.78–1.44)	1.29 (0.91–1.83)	97 (17.6)	0.78 (0.61–1.00)*	0.89 (0.67–1.18)
	Army	188 (7.8)	1	1	385 (21.5)	1	1
	RAF	51 (6.0)	0.80 (0.58–1.11)	1.09 (0.75–1.58)	119 (17.8)	0.79 (0.63–0.99)*	0.99 (0.76–1.29)
Deployment to Iraq/Afghanistan	Did not deploy	113 (7.8)	1	1	174 (17.9)	1	1
	Deployed	189 (13.1)	0.87 (0.68–1.11)	1.02 (0.76–1.36)	427 (21.0)	1.22 (1.01–1.49)*	1.08 (0.86–1.35)
Role in parent unit	Combat support/CSS	183 (6.4)	1	1	395 (18.2)	1	1
	Combat	112 (10.1)	1.61 (1.26–2.06)***	1.10 (0.81–1.51)	204 (24.7)	1.48 (1.22–1.79)***	1.22 (0.97–1.53)
Method of leaving	Planned	179 (5.2)	1	1	465 (17.7)	1	1
	Unplanned	30 (19.7)	4.59 (2.98–7.08)***	3.04 (1.85–5.00)***	31 (36.5)	2.67 (1.70–4.20)***	2.17 (1.33–3.54)**
	Medical	69 (25.9)	6.75 (4.90–9.28)***	6.40 (4.50–9.09)***	80 (40.6)	3.18 (2.35–4.30)***	2.50 (1.80–3.48)***

* *p* < 0.05 ** *p* < 0.01 *** *p* < 0.001

^a^Adjusted for all factors except sex and deployment

^b^Adjusted for all factors except sex

## Discussion

The main findings were that method of leaving, especially medical discharge, had a strong association with negative socioeconomic outcomes, especially unemployment, and also with mental ill health, particularly PTSD. Another consistent predictor of negative outcomes (with the exception of unemployment) was childhood adversity. Lower rank was also frequently associated with these negative outcomes, as was younger age and lower education. Few of the members of this cohort, who had been in active service during the Iraq War or later, were under 30 years of age. Younger leavers were at higher risk of alcohol misuse (a phenomenon also observed among US veterans [23]), and of unemployment and financial hardship. The majority of the Armed Forces are male, which was associated with alcohol misuse.

Prior research on UK leavers found that men, ex-RAF, and NCOs were more likely to find employment after leaving [24]; this study largely confirms those findings, as we also found out that higher rank was protective against unemployment while service in the Army increased the risk. Our initial finding that women were more likely to be unemployed was explained by maternity, which may explain the lower rate of post-Service re-employment among women in the prior study. Although rank and childhood adversity were not associated with unemployment, they were associated with financial difficulty. This suggests that veterans from these groups may not have difficulty finding re-employment per se but the jobs they find may have low status and pay.

Previous analysis of veterans using this sample found that combat role was associated with probable PTSD but not alcohol [13], findings which are supported by this study. One difference between these studies is that, after adjustment, this study did not find an association between combat role and CMD; this may be because this study is more inclusive and was more stringent in relation to adjustment for possible confounders.

Many of the factors found to be associated with negative transition outcomes in our study, including mental health outcomes, have previously been found to be associated with leaving Service early [25]; this suggests that the higher rate of mental health disorders among veterans compared with serving personnel reported previously [13] is a topic deserving careful consideration. It may be the case that the higher rates of veteran mental ill health are the consequences of poor mental health causing early exit and subsequent difficulty in transition due to a lack of planning and preparation. Alternative explanations might be a change in self-appraisal of health after leaving the Forces; or a stressful transition which may result in an increase in mental ill health symptoms.

Most leave either at the end of their term of service, or voluntarily; however, of the minority that leave in an unplanned way, many receive medical discharges. These unplanned and medical leavers had worse post-service outcomes by all measures; furthermore, these associations had generally larger effect sizes than other factors. Medical dischargees were usually the worst-affected group, though this may be partly due to medical issues acting as a barrier to re-employment. The exception was among the alcohol misuse group in which exit was not related to medical but to other unplanned discharge (which may be a consequence of misconduct and disciplinary offences). This finding is important because it indicates that uncontrolled drinking behaviour may contribute to premature discharge from the services.

Overall this analysis suggests that military experiences play a lesser role than socio-demographic factors when it comes to post-service outcomes; whilst it may be argued that the observed effects of rank indicate that differing military experiences impact veteran outcomes, rank is itself indicative of socioeconomic status [26], suggesting that this association partially arises from socioeconomic circumstances or background. These findings suggest that support for veterans into civilian life could be provided equally or even preferentially for unplanned leavers (particularly Ministry of Defence-mandated resettlement which has historically provided more support for longer servers and limited support for unplanned leavers), and medical dischargees should be actively followed-up to ensure they are making use of the full support available to them.

Our findings highlight a fundamental policy dilemma. While policy regarding transition has changed, e.g. introducing a programme for early Service leavers, it is still the case that transition and resettlement provisions become increasingly generous with longer service, yet the need for these provisions becomes progressively less necessary as personnel acquire seniority and skills. On the one hand politicians and the public almost certainly will instinctively agree that longer service should be rewarded. But the alternative argument can be made, that this should be on the basis of need (and take into account the needs of and risk factors present in the individual) and its potential effectiveness.

### Strengths and limitations

Study respondents were originally selected while still serving in the UK Armed Forces; thus, this is effectively a prospective cohort. This avoids the limitations of identifying and reaching a retrospective group of veterans, which is utilised in most other studies on veterans and has a high risk of being unrepresentative. The original sample was generated to be representative of the makeup of two sampling frames from the UK Armed Forces in 2003, those deployed to Iraq and those not deployed to Iraq (at that time). The sample of veterans here, which was non-random as it was a sub-sample defined by the event of leaving and hence analysed without weighting, should nonetheless be approximately representative of veterans who left between 2003 and 2016. It is unlikely that any departure from representativeness would not have biased any of the associations reported in this study. Compared with MoD transition surveys, this study covers a much wider period of time post-service and represents a sample that includes the full spectrum of the trained strength of the Armed Forces in contrast to the limited selective sample of those who undertook resettlement activities [27]. As this study uses data from several phases of data collection, not all results are contemporaneous, but this has the advantage of presenting outcomes over a broader range of time. Unplanned leavers are underrepresented in this study, as they may drop out of contact in the time between selection into the study and sampling. It was not possible to determine time of onset of mental health problems, as in most population-based studies; the analysis is of prevalent cases of symptoms post-service, which includes both new and old cases of the disorders being explored. This issue also prevents longitudinal analyses. The information was reported by the participants and we cannot exclude the possibility of recall bias.

## Conclusion

Certain groups appear to be at risk of poor mental health and socio-economic hardship after leaving the Services; factors related to socio-economic status are consistently associated with such hardship. Method of leaving had a strong effect, suggesting that the greatest positive impact might be to increase support for unplanned leavers, in particular those who leaving with a medical discharge (some of whom will be discharged due to mental health), who have substantially worse outcomes by all measures, though unplanned leave on its own also increases the risk of mental disorder and socio-economic hardship. Support provision at present may reflect a public desire to give greater rewards for greater service and focuses solely on military experiences while greater impact in improving transition outcomes may be achieved by addressing underlying sociodemographic factors. Ultimately this is a social, political and ethical dilemma.

## Data Availability

The datasets generated and analysed during the study are not publicly available due to the sensitive nature of data referring to members of the Armed Forces.
